# Late-onset OHVIRA syndrome in a 49-year-old woman with severe dysmenorrhea: a case report on vaginoscopic management

**DOI:** 10.3389/fmed.2026.1769370

**Published:** 2026-02-25

**Authors:** Yusuf Ziya Kizildemir, Ömer Tammo, Mehmet İncebiyik, Muhammed Erdal Sak, Sibel Sak

**Affiliations:** Department of Gynaecology and Obstetrics, Faculty of Medicine, Harran University, Sanliurfa, Türkiye

**Keywords:** late-onset presentation, Müllerian duct anomalies, OHVIRA syndrome, uterus didelphys, vaginoscopic resection

## Abstract

OHVIRA syndrome is a rare Müllerian duct anomaly characterized by uterus didelphys, obstructed hemivagina, and ipsilateral renal agenesis. Although typically diagnosed in adolescence, it can rarely present later in life. We present the case of a 49-year-old woman who presented with severe dysmenorrhea and intermittent spotting. Imaging studies, particularly MRI, confirmed the diagnosis of OHVIRA syndrome by revealing a didelphic uterus with hematometra and hematocolpos secondary to a right-sided obstructed hemivagina, along with right renal agenesis. The patient was successfully managed with vaginoscopic and hysteroscopic resection of the vaginal septum. Postoperatively, her symptoms resolved completely, with no recurrence observed at 6-month follow-up. This case contributes to the literature by documenting one of the latest known presentations of OHVIRA syndrome, reinforcing that a high index of suspicion for Müllerian anomalies must be maintained regardless of patient age. It demonstrates that a partially draining orifice can mask classic symptoms for decades and confirms the long-term efficacy of a minimally invasive surgical approach for both diagnosis and treatment.

## Introduction

OHVIRA syndrome is a rare Müllerian duct anomaly characterized by the coexistence of a didelphic uterus, obstructed hemivagina, and ipsilateral renal agenesis (1). Although the true incidence of OHVIRA syndrome remains uncertain, its prevalence has been reported to range between 0.1 and 3.8% (2). Patients typically present with symptoms such as dysmenorrhea, lower abdominal pain, pelvic mass, urinary retention, and frequent pelvic or urinary infections, secondary to the development of hematometra and hematocolpos after puberty and menarche (3). While most cases are identified shortly after menarche, a growing number of reports have described diagnoses in women in their 20s and 30s. However, a diagnosis approaching the fifth decade of life, as in our patient, remains an exceptional finding in the existing literature (2). Here, we present the case of a 49-year-old woman with OHVIRA syndrome, highlighting the diagnostic challenges of a late presentation and the efficacy of minimally invasive surgical management.

## Case report

**Figure 1:**
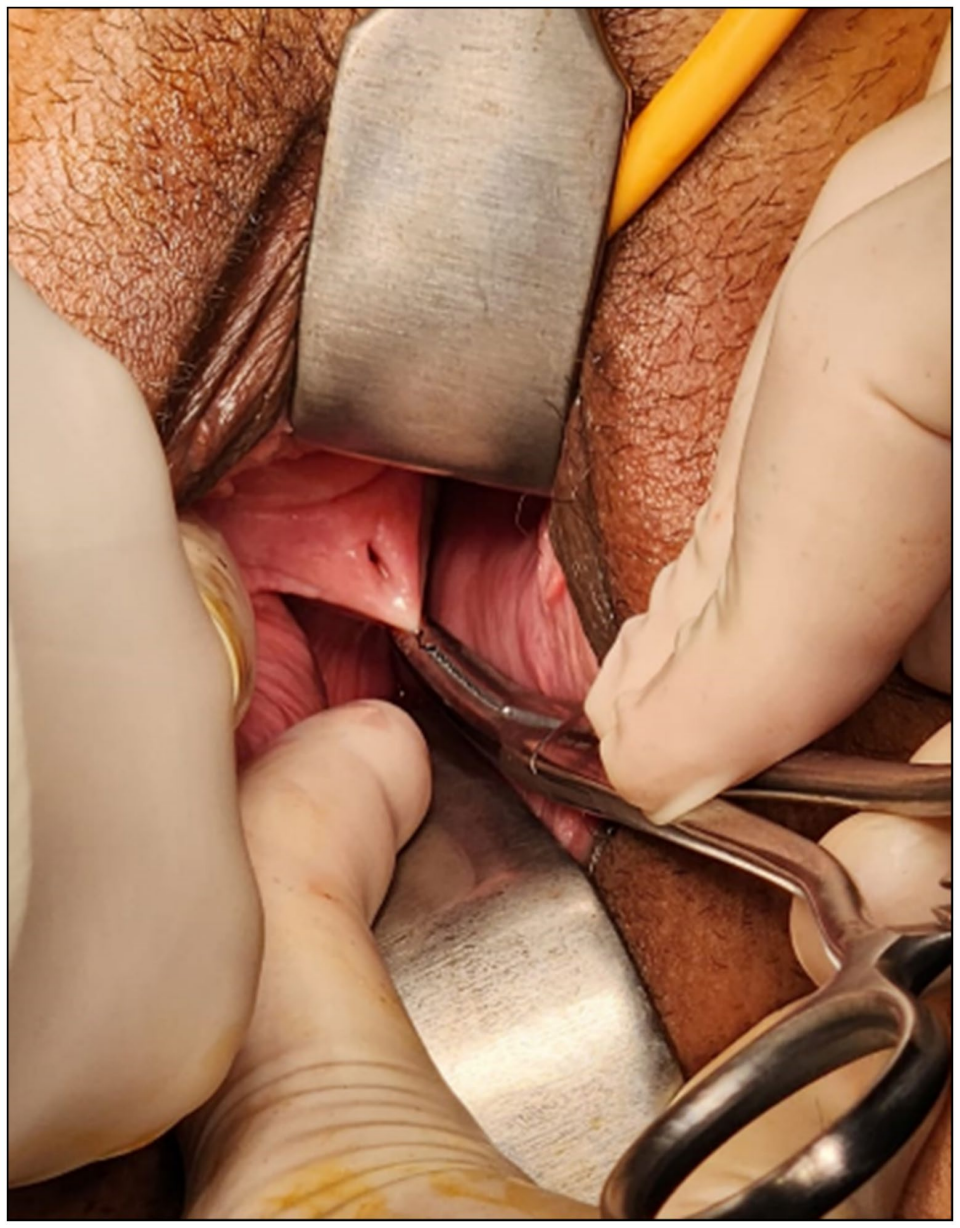
Small obstructed drainage hole in the right vaginal wall.

Written informed consent was obtained from the patient for the publication of this case report and any accompanying images. A 49-year-old woman presented with a history of severe dysmenorrhea, intermittent lower abdominal pain associated with vaginal spotting, and brownish discharge. Having started menstruating at the age of 12, the patient had regular menstrual cycles with a normal flow but suffered severe dysmenorrhea without menorrhagia. She described intermenstrual spotting, brownish discharge between menstrual periods, and abnormal vaginal discharge. The patient reported that these chronic and debilitating symptoms had significantly impacted her quality of life, causing considerable distress and interfering with her daily activities. Bowel and bladder habits were normal. Her obstetric history was significant for three miscarriages and six preterm deliveries before 36 weeks of gestation. All deliveries were spontaneous vaginal births without malposition or malpresentation. Because they were vaginal deliveries, the vaginal septum and uterine anomaly remained undiagnosed at that time. Currently, all six of her children are alive and healthy. Additionally, the patient had a history of abdominal surgery due to bowel perforation, resulting in midline incisions both below and above the umbilicus. Physical examination revealed normal secondary sexual characteristics and external genitalia. Vaginal examination revealed a single cervix shifted to the left and a palpable mass in the right fornix. A small obstructed drainage orifice was observed on the right vaginal wall (Figure 1). The orifice was found to be blocked. The patient reported that this orifice had previously provided partial drainage and temporary relief of her symptoms before it became obstructed.

**Figure 2:**
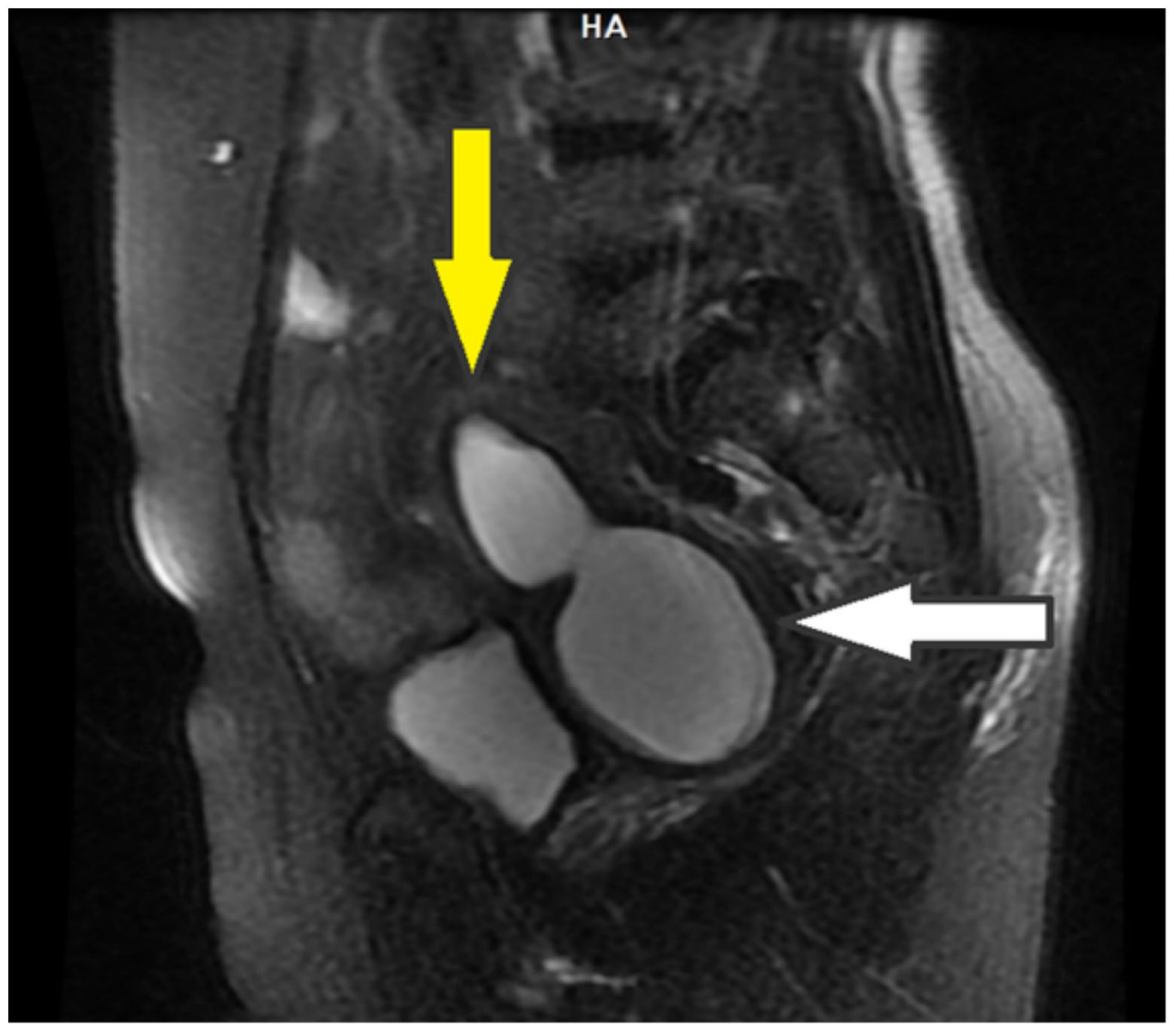
MRI: Sagittal T2-weighted image shows the presence of hematocolpos (white arrow) and hematometra (yellow arrow).

**Figure 3:**
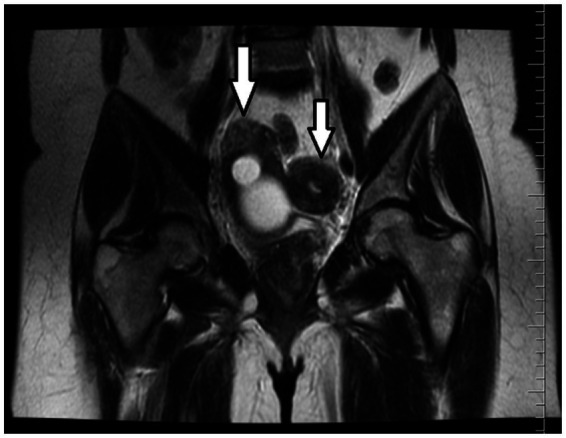
MRI: Coronal T2-weighted image shows the presence of two uterine bodies (white arrows).

**Figure 4:**
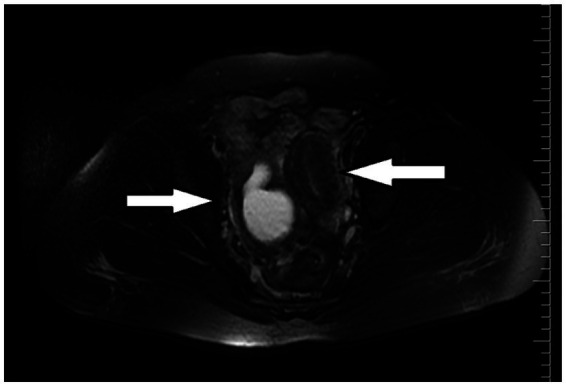
MRI: Axial T2-weighted image shows the presence of two uterine bodies (white arrows).

An initial ultrasound performed at an outside center was interpreted as a complex right adnexal mass. Given her symptoms, the initial differential diagnosis included a tubo-ovarian abscess, an endometrioma, or a complex ovarian cyst. However, the mass’s location and the physical examination findings at our center suggested a Müllerian anomaly. To clarify the diagnosis, a pelvic MRI was performed. The MRI was definitive, demonstrating a didelphic uterus with significant hematometra and hematocolpos in the right hemiuterus, consistent with a right-sided obstructed hemivagina. As Müllerian anomalies are often associated with urinary tract defects, a renal evaluation was conducted, which confirmed right renal agenesis with compensatory hypertrophy of the left kidney. These collective findings established the classical diagnosis of right-sided OHVIRA syndrome (Figures 2–4). Except for a high C-reactive protein (CRP) level of 45.6 mg/dL, no other specific features were observed in the blood test. Hemoglobin levels were 11 g/dL. Serology and screening for sexually transmitted infections were negative, and tumor markers were within normal ranges.

The patient’s clinical course followed this timeline:

1. Age 12: Onset of menarche, with regular cycles but severe dysmenorrhea.
2. Ages 20–40 (approximately): A long history of intermenstrual spotting and brownish discharge, partially alleviated by a small drainage orifice. Obstetric history was marked by three miscarriages and six preterm deliveries.
3. Age 49 (presentation): The drainage orifice became fully obstructed, leading to symptom exacerbation and presentation to our clinic.
4. Initial diagnosis (outside center): Ultrasound was misinterpreted as a right adnexal mass.
5. Definitive diagnosis (our center): Pelvic examination and subsequent MRI confirmed uterus didelphys, obstructed right hemivagina with hematometra and hematocolpos, and ipsilateral renal agenesis.
6. Intervention: The patient underwent successful vaginoscopic and hysteroscopic resection of the vaginal septum.
7. Follow-up (6 months): The patient was asymptomatic with complete resolution of hematometra and hematocolpos confirmed by MRI.

**Figure 5:**
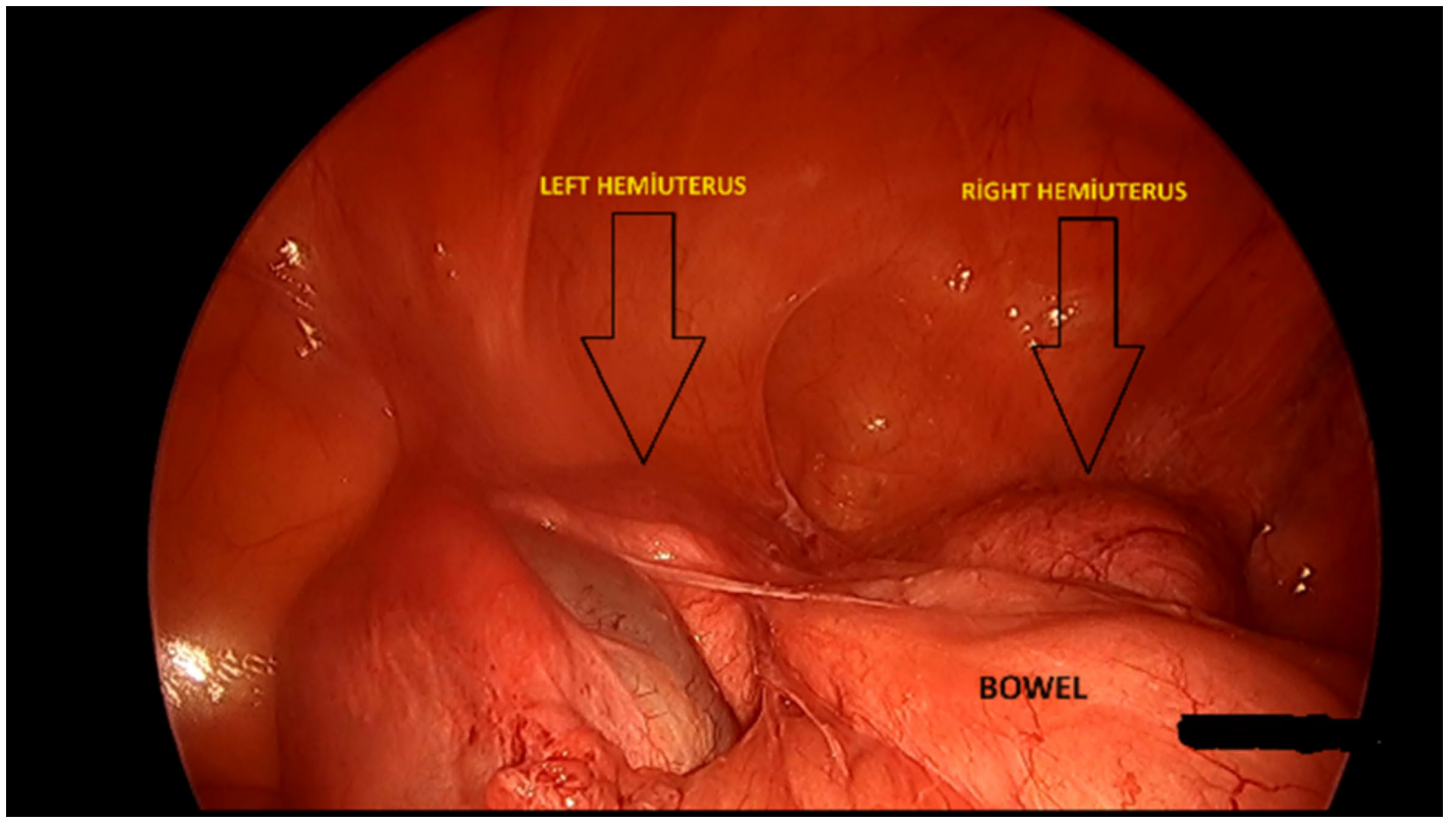
Laparoscopic image of didelphys uterus and pelvic adhesions.

**Figure 6:**
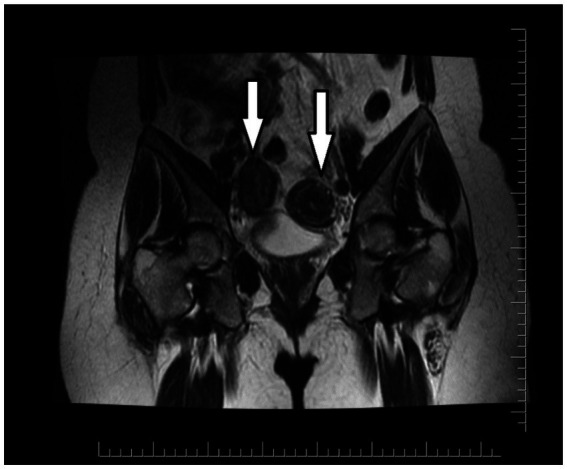
MRI: Coronal T2-weighted image shows the presence of a normal didelphic uterus at the 6-month follow-up (white arrows).

**Figure 7:**
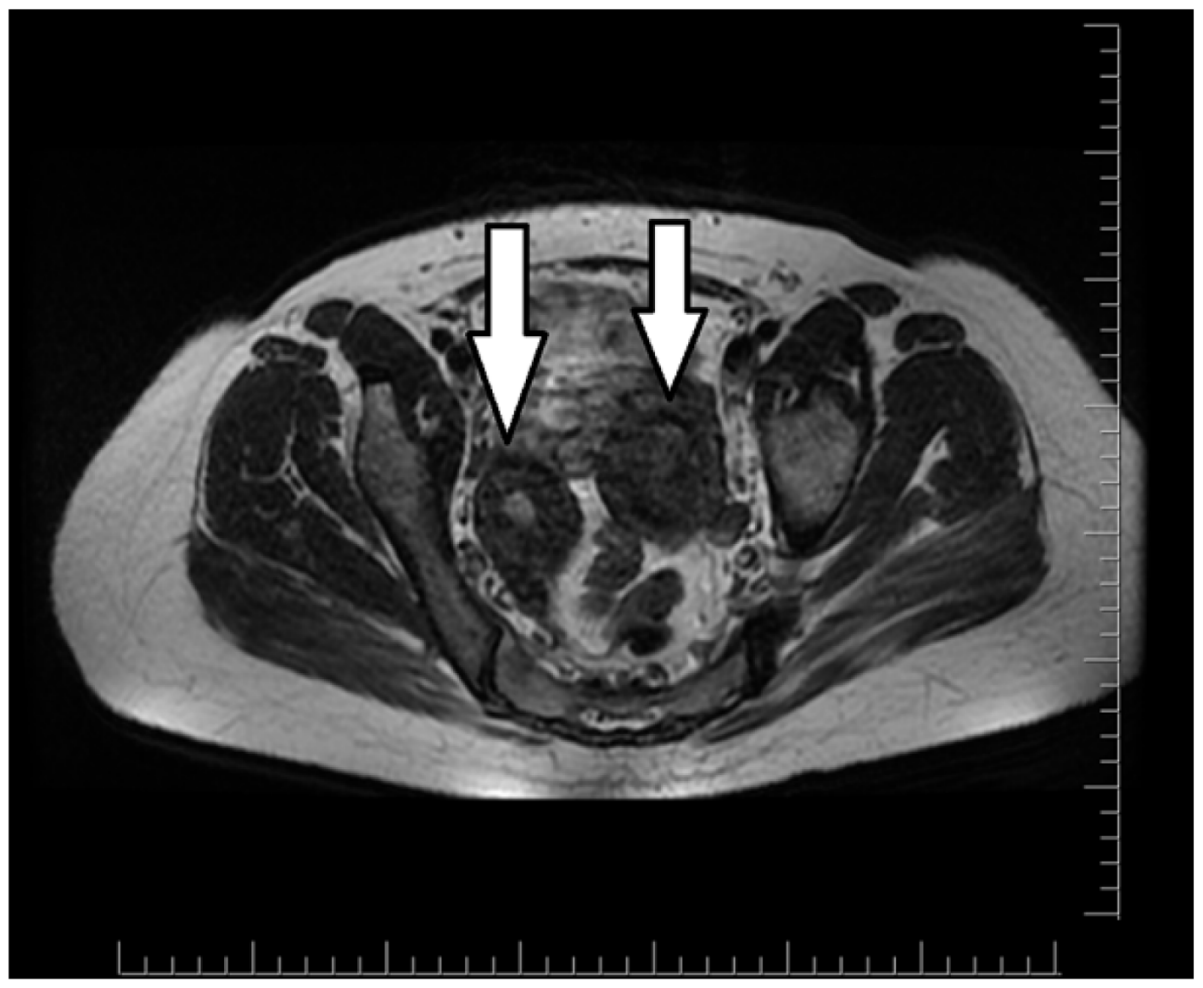
MRI: Axial T2-weighted image shows the presence of a normal didelphic uterus at the 6-month follow-up (white arrows).

Vaginoscopic vaginal septum incision under anesthesia was planned for the patient. Informed consent was obtained for the surgery. Laparoscopy confirmed the diagnosis of didelphic uterus. The primary goal of laparoscopy was to confirm the anatomy and simultaneously identify and treat potential endometriosis. During the same sitting, pelvic endometriotic foci were cauterized, and adhesions were adhesiolysed to alleviate chronic pelvic pain. No hematosalpinx was observed. Adhesions and endometriosis, attributed to retrograde menstruation, were identified in the pelvic region (Figure 5). Vaginoscopy revealed a single cervix at 1 o’clock and a lump at 10 o’clock on the right side. Hysteroscopic examination of the left uterine cavity and ostium appeared normal. An oblique incision of 2 cm was made over the lump on the right side of the cervix using a resectoscope. Drainage was facilitated through the newly created opening. Hysteroscopy revealed a normal right uterine cavity and ostium. To ensure the continuity of drainage, a No. 20 silicone Foley catheter was placed from the septal opening to the right vaginal cavity. The postoperative period was uneventful and painless. On the first postoperative day, ultrasound examination revealed the complete resolution of hematometra and hematocolpos. The patient was discharged with an *in situ* catheter and oral contraceptives for 2 weeks to prevent the newly created vaginal opening from occlusion. Due to the presence of pelvic endometriosis and the patient’s ongoing menstrual status, she was subsequently started on Dienogest (2 mg/day) to suppress endometriotic activity and prevent symptom recurrence until she reaches menopause. During the 6-month follow-up, no recurrence of hematometra or hematocolpos was observed, indicating the efficacy of the surgical treatment and its long-term success (Figures 6, 7). The use of minimally invasive techniques such as vaginoscopy and hysteroscopy in surgical intervention reduced the risk of complications and expedited the patient’s postoperative recovery.

## Discussion

This case report presents an exceptionally rare instance of OHVIRA syndrome diagnosed in a 49-year-old woman, making it one of the latest-onset cases documented in the literature. While most cases are diagnosed in the second decade, our case at 49 years old stands as an outlier, similar to only a handful of cases reported globally in perimenopausal women (2). The syndrome, with a prevalence estimated between 0.1 and 3.8% among women with uterine anomalies (2), typically presents in adolescence shortly after menarche. While the systematic review by Bonetti et al. confirms that most diagnoses occur before age 30 (2), our patient’s presentation in her fifth decade is a significant deviation from the norm. This late diagnosis highlights challenges posed by atypical clinical features, particularly the presence of a partially draining septum (4).

The primary reason for the decades-long delay in diagnosis was the presence of a small, partially draining orifice in the vaginal septum. This fenestration allowed for intermittent drainage of menstrual blood, preventing the formation of a large, acutely symptomatic hematocolpos that would typically prompt earlier investigation. Consequently, the patient’s symptoms were chronic severe dysmenorrhea and intermenstrual brownish discharge rather than the acute, cyclical pelvic pain commonly seen in adolescents. This clinical scenario, where an incomplete obstruction alters the classical presentation, is well-documented in the literature. For instance, Wozniakowska et al. reported a case of Herlyn–Werner–Wunderlich syndrome in a 14-year-old whose diagnosis was delayed due to a similar microperforation (4). In their patient, this small fistula resulted in atypical symptoms of pyocolpos and chronic purulent discharge rather than a classic hematocolpos. Although their case was in an adolescent, it demonstrates the crucial underlying mechanism: a partial drainage pathway can effectively mask the signs of complete obstruction for a prolonged period.

Imaging plays a pivotal role in diagnosing complex Müllerian duct anomalies. While ultrasound is a valuable initial tool, it can be misleading, as evidenced by the initial interpretation of a right adnexal mass in this case. Magnetic resonance imaging (MRI) is the gold standard, offering superior anatomical detail essential for accurate diagnosis and surgical planning (5, 6). In our patient, MRI was indispensable, clearly demonstrating the didelphic uterus, the obstructed right hemivagina with hematometra and hematocolpos, and the associated ipsilateral renal agenesis, thereby confirming the classical findings of OHVIRA syndrome (7).

The definitive management of OHVIRA syndrome is the surgical resection of the obstructing vaginal septum to ensure adequate drainage and resolve symptoms. We used a minimally invasive vaginoscopic and hysteroscopic approach, which offers advantages such as reduced morbidity and faster recovery (8). The complete resolution of the patient’s symptoms and the absence of recurrence at the 6-month follow-up confirm the long-term efficacy of this approach. Laparoscopy, while not therapeutic for the septum itself, was crucial for confirming the uterine anomaly and identifying co-existing pathologies such as endometriosis and pelvic adhesions, which are common sequelae of chronic retrograde menstruation and can lead to infertility if left untreated (9).

The primary *strength* of our management was the use of a combined vaginoscopic and laparoscopic approach, which allowed for both the relief of the obstruction and the treatment of secondary endometriosis in a single, minimally invasive session. However, a *limitation* of this case is the 49-year delay in diagnosis, which likely contributed to the patient’s poor obstetric history and decades of preventable chronic pain.

A significant aspect of this case is the patient’s poor obstetric history, which included three miscarriages and six preterm deliveries. While a definitive causal link cannot be proven retrospectively, it is reasonable to hypothesize that the undiagnosed OHVIRA syndrome directly contributed to these adverse outcomes. The anatomical abnormalities inherent to uterus didelphys, a core component of this syndrome, are strongly associated with such complications. Factors including reduced uterine cavity volume in each hemiuterus, potential cervical incompetence, and abnormal uterine vasculature are known to impair implantation and the ability to carry a pregnancy to term, leading to an increased risk of miscarriage and preterm delivery (1, 10, 11). This case, therefore, reinforces that Müllerian anomalies should be considered in the differential diagnosis of recurrent pregnancy loss and preterm delivery, even in patients with seemingly regular menstruation.

## Conclusion

This case demonstrates that OHVIRA syndrome can remain undiagnosed until the fifth decade, particularly when atypical symptoms are masked by a partially draining orifice. Clinicians must maintain a high index of suspicion for Müllerian duct anomalies in patients of any age presenting with chronic dysmenorrhea, a pelvic mass, or a poor obstetric history, regardless of menstrual regularity. Advanced imaging, particularly MRI, is essential for accurate diagnosis, while minimally invasive surgical resection of the vaginal septum is a safe and effective treatment for long-term symptom relief. For patients desiring future fertility, a laparoscopic evaluation for associated endometriosis or adhesions is critical. Postoperative follow-up should include imaging to confirm sustained drainage and clinical assessment to ensure the resolution of symptoms.

## Data Availability

The original contributions presented in the study are included in the article/supplementary material, further inquiries can be directed to the corresponding author.
